# Individualized post-crisis monitoring of psychiatric patients via Hidden Markov models

**DOI:** 10.3389/fdgth.2024.1322555

**Published:** 2024-02-02

**Authors:** Roger Garriga, Vicenç Gómez, Gábor Lugosi

**Affiliations:** ^1^ Koa Health, Barcelona, Spain; ^2^ Department of Information and Communication Technologies, Universitat Pompeu Fabra, Barcelona, Spain; ^3^ ICREA, Barcelona, Spain; ^4^ Department of Economics and Business, Universitat Pompeu Fabra, Barcelona, Spain; ^5^ Barcelona School of Economics, Barcelona, Spain

**Keywords:** probabilistic modeling, mental health crisis, Hidden Markov model, mental health, psychiatry, machine learning, predictive analytics

## Abstract

**Introduction:**

Individuals in the midst of a mental health crisis frequently exhibit instability and face an elevated risk of recurring crises in the subsequent weeks, which underscores the importance of timely intervention in mental healthcare. This work presents a data-driven method to infer the mental state of a patient during the weeks following a mental health crisis by leveraging their historical data. Additionally, we propose a policy that determines the necessary duration for closely monitoring a patient after a mental health crisis before considering them stable.

**Methods:**

We model the patient’s mental state as a Hidden Markov Process, partially observed through mental health crisis events. We introduce a closed-form solution that leverages the model parameters to optimally estimate the risk of future mental health crises. Our policy determines a patient should be closely monitored when their estimated risk of crisis exceeds a predefined threshold. The method’s performance is evaluated using both simulated data and a real-world dataset comprising 162 anonymized psychiatric patients.

**Results:**

In the simulations, 96.2% of the patients identified by the policy were in an unstable state, achieving a F1 score of 0.74. In the real-world dataset, the policy yielded an F1 score of 0.79, with a sensitivity of 79.8% and specificity of 88.9%. Under this policy, 67.3% of the patients should undergo close monitoring for one week, 21.6% during 2 weeks or more, while 11.1% do not need close monitoring.

**Discussion:**

The simulation results provide compelling evidence that the method is effective under the specified assumptions. When applied to actual psychiatric patients, the proposed policy showed significant potential for providing an individualized assessment of the required duration for close and automatic monitoring after a mental health crisis to reduce the relapse risks.

## 1 Introduction

A mental health crisis is any situation in which a person’s behavior puts them at risk of hurting themselves or others and/or prevents them from being able to care for themselves or function effectively in the community (1). Those situations include self-harm, delusions or suicide attempts, often requiring hospitalization, and are very detrimental to the patient’s mental and social wellbeing. Mental health crises are commonly suffered by patients diagnosed with psychotic, personality or severe mood disorders. However, they also occur to patients diagnosed with less severe disorders or even non diagnosed individuals under stressful situations (1). The patient usually undergoes four phases in the process of a crisis (2), (i) an initial threat when the patient is stable, (ii) an escalation phase, (iii) the crisis (iv) resolution and return to stability or personality disorganization if the problem does not get resolved. Once the patient destabilizes, they remain unstable for some period of time during which they might suffer one or multiple mental health crises. In order to avoid further escalation and prevent subsequent crises, patients should be kept under close monitoring and treatment until they stabilize (3–6). However, it is difficult to ascertain when the patient has become stable.

In this work, we present a data-driven method to infer the mental state of a patient given their history of mental health crises and propose a policy to determine for how many weeks the patient needs to receive close attention before being deemed stable. This method is based on modeling the mental state of the patient as a Hidden Markov Model (HMM) (7), a probabilistic framework in which the observed data is generated by one or multiple hidden states. This allows one to infer whether the patient is stable or unstable and make a prediction of the risk that the patient is going to suffer a mental health crisis. Under this modeling framework, our method is implemented in the following way:

1. **Learning the model parameters of each patient:** Initially, the model parameters for the average patient are determined by maximizing the likelihood of the observed sequence of mental health crises experienced by all patients. These parameters are assigned to patients with a relatively short history at the hospital (3 months or less in this study). For patients with a longer history, the model parameters of each patient are estimated from their individual observed sequence of mental health crises.
2. **Estimating the risk of mental health crisis at each week:** For a given patient, the risk is estimated based on the patient’s model parameters, taking into account the time elapsed since their last mental health crisis.
3. **Selecting the patients to be closely monitored:** Identify those patients whose predicted risk of a mental health crisis exceeds a predefined threshold. Patients that do not reach the threshold are considered stable due to their low risk to suffer a mental health crisis.

Probabilistic models are very well suited to uncover hidden phenotypes or internal states in healthcare settings and to build policies based on partial observability of internal states (8). The use of HMM’s to infer the mental state of an individual has been explored in the past for detecting depressive states or schizophrenic episodes (9–11) and identifying mental disorders (12, 13). A similar probabilistic model called maximum-entropy Markov model was used to predict emergency psychiatric states (14) from biometric sensors and questionnaires. However, these studies rely on external data sources such as sensor data, questionnaires or other user inputs. In contrast, our work proposes a method that relies solely on past crises to infer the mental state of the patient, which is accessible in any hospital and does not require external data collection.

Previous research has demonstrated the feasibility of predicting mental health crisis relapses when patients appear stable utilizing a machine learning model based on Electronic Health Records (EHR) (15, 16). However, these studies assumed that all patients achieved stability after just one week without crisis events. In reality, certain patients might necessitate prolonged and vigilant monitoring to ascertain their stability accurately and avoid readmission. The present study complements the existing literature by introducing a method to determine the optimal duration of monitoring required for each individual patient before they can be confidently deemed stable. By adopting this data-driven approach, clinicians can make informed decisions that facilitate personalized care. This approach follows the principle of Precision Medicine, a field that has been implemented across various healthcare domains and is now gaining traction within the field of psychiatry, promising enhanced patient outcomes and more effective interventions (17, 18).

## 2 Materials and methods

In this section, we detail the steps required to implement our proposed method. In Section 2.1, we formalize the problem and the mental state model upon which our method is built, and discuss the assumptions. In Section 2.2, we present an optimal solution to predict the risk that a patient will suffer a mental health crisis within the next week given the model parameters, and how the risk evolves over time. In Section 2.3, we describe the process to estimate the model parameters from a sequence of weeks with and without mental health crisis events. Finally, in Section 2.4, we propose a policy for determining the duration a hospital should closely monitor patients before deeming them stable.

### 2.1 Mental health state probabilistic model

We consider a hospital with N patients, with each patient n having an associated mental health state $X_{t,n}\in \{S,U\}$ at each week t=0,…,T and a binary random variable $Y_{t,n}$ that denotes whether the patient had a mental crisis at week t ($Y_{t,n}=1$) or not ($Y_{t,n}=0$). Every week t of the patient n is characterised by the $\left(X_{t,n},Y_{t,n}\right)$ pair and we denote by $H_{t,n}=\{(X_{0,n},Y_{0,n}),\ldots ,$ $\left(X_{t,n},Y_{t,n})\right\}$ the entire history of the patient up to week t. We use $x_{t,n}$ and $y_{t,n}$ to denote the realizations of $X_{t,n}$ and $Y_{t,n}$, and introduce the notation $Y_{a,n}^{b}=(Y_{a,n},\ldots ,Y_{b,n})$, a<b∈Z (similarly for other random variables $X_{a,n}^{b}$ and realizations $x_{a,n}^{b}$).

#### 2.1.1 Assumptions

We consider the following set of assumptions associated with our problem:

• There are two possible mental states that a patient n can have at any week t, stable (S) or unstable (U), thus $X_{t,n}\in \{S,U\}$, ∀t=1,…,T.
• The mental state of the patient evolves following a Markov Chain (i.e., $P(X_{t,n}=x_{t,n}|X_{0,n}=x_{0,n},\ldots ,X_{t-1,n}=x_{t-1,n})=$ $P(X_{t,n}=x_{t,n}|X_{t-1,n}=x_{t-1,n})$). We denote by $q=P(X_{t,n}=U|$ $X_{t-1,n}=S)$ the transition probability from state S to state U and by $r=P(X_{t,n}=U|X_{t-1,n}=U)$ the transition probability from state U to state U, with q≠r. This results in the following transition matrix:
  $$P_{X}=\left(\begin{array}{cc} \;p_{SS} & p_{SU}\\ p_{US} & p_{UU} \end{array}\right)=\left(\begin{array}{cc} 1-q & q\\ 1-r & r \end{array}\right)$$
• The probability that the patient n has a mental health crisis at time t depends solely on the state $X_{t,n}$. In particular, we assume that the patient cannot suffer a mental health crisis when the patient is at state S and when the patient is at state U the probability of crisis if 0<p<1, that is,
  $$P(Y_{t,n}=1|X_{t,n}=x_{t,n})=\left\{ \begin{array}{cc} 0 & \text{if }x_{t,n}=S\\ p & \text{if }x_{t,n}=U \end{array}\right.$$
• The actual mental state of the patient is hidden and only partially observed through the crisis variable. We denote by $O_{t,n}=\{Y_{0,n},\ldots ,Y_{t,n}\}$ the observed history up to time t.

We present two cases that depend on whether the patients of the hospital are characterized by homogeneous or diverse model parameters.

• Case 1: Each patient has a different set of model parameters.
• Case 2: All patients have the same set of model parameters.

### 2.2 Mental health crisis prediction

The purpose of the method is to predict whether a patient n is going to have a mental health crisis at time t given the observed history up to time t−1, $O_{t-1,n}$. In particular, we want to estimate the probability that $Y_{t,n}=1$ given $O_{t-1,n}$. Considering that the model parameters are known (or have been estimated as we will see in the next section), we can make inference on the current state of the patient given the observed history $O_{t-1,n}$ and use it to estimate the probability that the patient is going to have a crisis at time t. Since the prediction is done for each patient independently to the rest of the patients, we simplify the notation in this section by removing the subscript n.

First, we consider the case in which the last state $X_{t-1}$ is observed ($X_{t-1}\in O_{t-1}$). Using the Markov property we obtain that

$$\begin{array}{rl} P(Y_{t}=1|O_{t-1}) & =P(Y_{t}=1|X_{t}=U)P(X_{t}=U|O_{t-1})\\ & =P(Y_{t}=1|X_{t}=U)P(X_{t}=U|X_{t-1})\\ & =\left\{ \begin{array}{cc} \;pr & \mathrm{if}X_{t-1}=U\\ pq & \mathrm{if}X_{t-1}=S \end{array}\right.. \end{array}$$

A priori, we assumed that the state is never observed. However, there might be cases in which it is possible to observe the state either directly or indirectly. For instance, by monitoring the patient, the clinical teams can infer whether the patient is at stable state or not. Importantly, there are two consequences that follow from the proposed model: first, when a mental health crisis is observed at a time t−1 ($Y_{t-1}=1$), we can infer that $X_{t-1}=U$ because a patient can only suffer a mental health crisis when they are unstable; second, since the state transition is Markov, if the state is known at s<t ($X_{s}=x_{s}$) and there are no other known states between s+1 and $X_{t-1}$, then the distribution over states at $X_{t-1}$ only depends on the observations between s and t−1 and $X_{s}=x_{s}$. Therefore, without loss of generality, we can assume that the last observed state is at s=0 because the observations prior to s do not influence the probability distribution of states beyond s conditioned on $X_{s}=x_{s}$ (by the Markov property) - we could redefine a new $t^{\prime }=t-s$.

The following theorem presents a function to estimate the risk of mental health crisis at each week t after the last observed state. This is particularly relevant because the occurrence of a mental health crisis reveals that the patient is in an unstable state, and the theorem enables the determination of the number of weeks until the patient likely regains stability.

**Theorem 1:** Let $O_{t-1}=\{X_{0}=x_{0},Y_{0}=y_{0},Y_{1}=0,\ldots ,$ $Y_{t-1}=0\}$ be the observed history of a patient n up to the week t and p,q,r the model parameters associated with the patient. Then, the probability that patient n suffers a mental health crisis at week t is given by

(1) $$\begin{array}{rl} & P(Y_{t}=1|O_{t-1})=\\ & 1-(1-pr+r-q)\frac{(y_{0}-y_{-})y_{+}^{t+1}-(y_{+}-y_{0})y_{-}^{t+1}}{(y_{0}-y_{-})y_{+}^{t}-(y_{+}-y_{0})y_{-}^{t}}, \end{array}$$

with

$$\begin{array}{rl} y_{+} & =\frac{1+\sqrt{1-4\frac{\left(r-q)(1-p\right)}{(1-pr+r-q{)}^{2}}}}{2},\\ y_{-} & =\frac{1-\sqrt{1-4\frac{\left(r-q)(1-p\right)}{(1-pr+r-q{)}^{2}}}}{2},\\ y_{0} & =\frac{2Rw_{x_{0}}-R}{2R+w_{x_{0}}-1}, \end{array}$$

where $R=\frac{\left(r-q)(1-p\right)}{(1-pr+r-q{)}^{2}}$, $w_{U}=\frac{1-pr}{1-pr+r-q}$ (when $x_{0}=U$) and $w_{S}=\frac{1-pq}{1-pr+r-q}$ (when $x_{0}=S$).

To create a policy that works for all patients on a weekly basis, we need to understand how the estimated risk of a patient n experiencing a mental health crisis changes over time. The analytical solution from the theorem is particularly useful for this purpose, as it allows us to study how the risk evolves and converges. The following corollary demonstrates the convergence of the solution.

**Corollary 1.1:** The optimal solution to estimate the risk that a patient with model parameters q,r,p converges to $1-(1-pr+r-q)y_{+}$ when t grows.

Due to the exponential convergence primarily driven by the $y_{+}$ term, the convergence of the solution is expected to be rapid. This rapid convergence guarantees that the estimated risk does not oscillate indefinitely but rather quickly stabilizes at a steady value. Together, the results of Theorem 1 and the Corollary 1.1 show that we can estimate the risk of mental health crisis analytically and determine the week when a patient is likely to reach a stable state.

The proofs of Theorem 1 and Corollary 1.1 can be found in Supplementary Appendix A.

### 2.3 Estimation of the model parameters

The HMM has 3 parameters (p,q,r), specifically:

• p: the probability of mental health crisis given that the patient is at state U.
• q: the transition probability from state S to state U.
• r: the transition probability from state U to state U.

To simplify the notation, in this section we introduce p(a)=P(A=a) to denote the probability that a random variable A takes the value a (e.g., $p(x_{t,n})=P(X_{t,n}=x_{t,n})$). Similarly, we use the same notation for joint probabilities and conditional probabilities (e.g., $p(y_{0,n}^{t},x_{t,n})=P(Y_{0,n}^{t}=y_{0,n}^{t},X_{t,n}=x_{t,n})$ or $p(y_{t+1,n}^{T}|x_{t,n})=p(Y_{t+1,n}^{T}=y_{t+1,n}^{T}|X_{t,n}=x_{t,n})$).

We use the Baum–Welch algorithm (19) to estimate the model parameters from the observed history of the patients. This method, is a standard algorithm that uses an Expectation–Maximization approach to find the parameters that maximize the expected likelihood of the observed data given the model HMM. The Baum–Welch algorithm is guaranteed to converge to a local optimum (20) and consists of the following steps:

1. **Initialization:** The parameters of the model are initialized either randomly or using some reasonable estimates. In this case, we initialize the parameters (p, q, r) at random.
2. **Expectation step:** In this step, the probabilities of being in each hidden state at each time step t given the current model parameters and the observed sequence $O_{t,n}$ are calculated. These probabilities are computed using the Forward-Backward algorithm, that consist of a forward function $\alpha (x_{t,n})=p(y_{0,n}^{t},x_{t,n})$ defined as the joint probability of the observed data up to time t, and a backward function $\beta (x_{t,n})=p(y_{t+1,n}^{T}|x_{t,n})$ defined as the conditional probability of the observed data from time t+1 given the hidden state at t. Here, we abuse notation in $\alpha (x_{t,n})$ and $\beta (x_{t,n})$ by omitting the dependence on $y_{0,n}^{t}$ and $y_{t+1,n}^{T}$ respectively.
3. **Maximization step:** In this step, the probabilities calculated in the Expectation step are used to update the model parameters to maximize the expected log-likelihood of the observed data. This involves adjusting the probability p of mental health crisis when the patient is at state U and the transition probabilities between hidden states (q, r).
4. **Iterate:** Steps 2 and 3 are repeated iteratively until a convergence criterion is met. In this case, convergence criteria is set to stop when the change between two consecutive iterations is below a certain tolerance $\left(10^{-5}\right)$ or until a maximum number of iterations are completed (100).

#### 2.3.1 Case 1: Parameter estimation per patient

To estimate the parameters of the model for a patient n ($p_{n}$, $q_{n}$, $r_{n}$), we want to find the values $q_{n}^{*}$, $r_{n}^{*}$, $p_{n}^{*}$ that maximize the likelihood of the observed history of the patient. Since we are estimating the parameters of the model, the likelihood and all the probability distributions are conditioned to the value of the parameters, i.e.,

$$\begin{array}{rl} L(y_{1,n}^{T}|q_{n},r_{n},p_{n}) & =\log p(y_{0,n}^{T}|q_{n},r_{n},p_{n})\\ & =\log\underset{x_{0,n}^{T}\in \{S,U{\}}^{T}}{\sum }p(x_{0,n}^{T},y_{0}^{T}|q_{n},r_{n},p_{n}). \end{array}$$

For simplicity, we drop $q_{n}$, $r_{n}$, $p_{n}$ and the subscript n from the notation in the following equations. Let’s start with the joint probability of each state $x_{t}$ for t<T given a set of parameters $q_{n}$, $r_{n}$, $p_{n}$ and the observed data $O_{n}=y_{0,n}^{T}$.

$$p(x_{t},y_{0}^{T})=p(y_{0}^{T}|x_{t})p(x_{t})=p(y_{0}^{t}|x_{t})p(x_{t})p(y_{t+1}^{T}|x_{t})=p(y_{0}^{t},x_{t})p(y_{t+1}^{T}|x_{t})=\alpha (x_{t})\beta (x_{t}),$$

with $\alpha (x_{t})=p(y_{0}^{t},x_{t})$ and $\beta (x_{t})=p(y_{t+1}^{T}|x_{t})$ being the forward and backward functions repectively. Both $\alpha (x_{t})$ and $\beta (x_{t})$ can be computed iteratively.

The process of computing the $\alpha (x_{0}),\ldots ,\alpha (x_{T})$ is called forward step and can be derived as follows:

$$\alpha (x_{t})=p(y_{0}^{t},x_{t})=\underset{x_{t-1}\in \{S,U\}}{\sum }\alpha (x_{t-1})p(x_{t}|x_{t-1})p(y_{t}|x_{t}),$$

with $\alpha (x_{0})=p(x_{0})p(y_{0}|x_{0})$.

$\beta (x_{0}),\ldots ,\beta (x_{T})$ are computed iteratively starting backwards, this process is called the backward step:

$$\beta (x_{t})=p(y_{t+1}^{T}|x_{t})=\underset{x_{t+1}\in \{S,U\}}{\sum }\beta (x_{t+1})p(y_{t+1}|x_{t+1})p(x_{t+1}|x_{t}),$$

with $\beta (x_{T})=1$ and $\beta (x_{T-1})=\underset{x_{T}\in \{S,U\}}{\sum }p(y_{T}|x_{T})p(x_{T}|x_{T-1})$.

Through these expressions, $\alpha (x_{t})$ and $\beta (x_{t})$ can be computed for all t=0,…,T (21). From $\alpha (x_{t})$ and $\beta (x_{t})$ we can compute the probability distribution of the hidden states given the observations as

(2) $$\gamma (x_{t})=p(x_{t}|y_{0}^{T})=\frac{\;p(x_{t},y_{0}^{T})}{p(y_{0}^{T})}=\frac{\alpha (x_{t})\beta (x_{t})}{p(y_{0}^{T})}=\frac{\alpha (x_{t})\beta (x_{t})}{\underset{x_{t}\in \{S,U\}}{\sum }\alpha (x_{t})\beta (x_{t})}.$$

To finish the Expectation step, we need to compute the probability distribution of the transitions given the observations:

(3) $$\xi (x_{t},x_{t+1})=p(x_{t},x_{t+1}|y_{0}^{T})=\frac{\;p(x_{t},x_{t+1},y_{0}^{T})}{p(y_{0}^{T})}=\frac{\alpha (x_{t})p(x_{t+1}|x_{t})p(y_{t+1}|x_{t+1})\beta (x_{t+1})}{\underset{x_{t},x_{t+1}\in \{S,U{\}}^{2}}{\sum }\alpha (x_{t})\beta (x_{t+1})p(x_{t+1}|x_{t})p(y_{t+1}|x_{t+1})}.$$

In both Equations 2 and 3 the denominators are computed by regularizing the numerator to convert it to probabilities. Observe that we abused notation by omitting the dependence on $y_{0}^{T}$ when we defined $\gamma (x_{t})$ and $\xi (x_{t},x_{t+1})$.

Once the probability distribution of the hidden states and the transition probabilities given the observed data are computed, we can use them in order to estimate the new set of parameters in the Maximization step. In particular, the estimated value for the parameter $q_{n}$, ${\hat{q}}_{n}$, can be calculated as the expected number of transitions from state S to state U divided by the expected number of transitions starting at state S

$${\hat{q}}_{n}=\frac{\overset{T-1}{\underset{t=0}{\sum }}\xi (x_{t,n}=S,x_{t+1,n}=U)}{\overset{T-1}{\underset{t=0}{\sum }}\gamma (x_{t,n}=S)}.$$

The estimated value ${\hat{r}}_{n}$ for the parameter $r_{n}$, can be computed as the expected number of transitions from state U to state U divided by the expected number of transitions starting at state U,

$${\hat{r}}_{n}=\frac{\overset{T-1}{\underset{t=0}{\sum }}\xi (x_{t,n}=U,x_{t+1,n}=U)}{\overset{T-1}{\underset{t=0}{\sum }}\gamma (x_{t,n}=U)},$$

and the estimated value for the parameter $p_{n}$, ${\hat{\;p}}_{n}$, can be estimated as the expected number of times at state U and observing a crisis divided by the expected number of times at state U,

$${\hat{\;p}}_{n}=\frac{\overset{T-1}{\underset{t=0}{\sum }}1_{y_{t,n}=1}\gamma (x_{t,n}=U)}{\overset{T-1}{\underset{t=0}{\sum }}\gamma (x_{t,n}=U)}.$$

By iterating over the Expectation and Maximization step the algorithm converges to a local maximum on the likelihood function.

#### 2.3.2 Case 2: Single parameter estimation for all patients

To estimate the parameters of the model assuming that all the patients have the same parameter values (p, q, r), we want to find the values $p^{*}$, $q^{*}$ and $r^{*}$ that maximize the likelihood of the observed history of all the patients. Since the observations of each patient are independent of the rest of the patients, we have

$$\begin{array}{rl} L(y_{0,1}^{T},\ldots ,y_{1,N}^{T}|q,r,p) & =\log p(y_{0,1}^{T},\ldots ,y_{0,N}^{T}|q,r,p)\\ & =\overset{N}{\underset{n=1}{\sum }}\log\underset{x_{0,n}^{T}\in \{S,U{\}}^{T}}{\sum }p(x_{0,n}^{T},y_{0}^{T}|q,r,p). \end{array}$$

Furthermore, the joint probabilities, given the parameters p, q and r, can be computed per patient independently. Therefore, the results of the expectation step derived in the previous section can be used to compute the probability distribution of the hidden states and the probability distribution of the transitions given the observations of the patient. In this case, we define $\alpha_{n}(x_{t,n})$, $\beta_{n}(x_{t+1,n})$, $\gamma_{n}(x_{t,n})$ and $\xi_{n}(x_{t,n},x_{t+1,n})$ for each patient n like in Case 1, which are computed as

$$\begin{array}{rl} \alpha_{n}(x_{t,n}) & =\underset{x_{t-1,n}\in \{S,U\}}{\sum }\alpha_{n}(x_{t-1,n})p(x_{t,n}|x_{t-1,n})p(y_{t,n}|x_{t,n}),\\ \beta_{n}(x_{t,n}) & =\underset{x_{t+1,n}\in \{S,U\}}{\sum }\beta_{n}(x_{t+1,n})p(y_{t+1,n}|x_{t+1,n})p(x_{t+1,n}|x_{t,n}),\\ \gamma_{n}(x_{t,n}) & =\frac{\alpha_{n}(x_{t,n})\beta_{n}(x_{t,n})}{\underset{x_{t,n}\in \{S,U\}}{\sum }\alpha_{n}(x_{t,n})\beta_{n}(x_{t,n})}.\\ \xi_{n}(x_{t,n},x_{t+1,n}) & =\frac{\alpha_{n}(x_{t,n})p(x_{t+1,n}|x_{t,n})p(y_{t+1,n}|x_{t+1,n})\beta_{n}(x_{t+1,n})}{\underset{x_{t,n},x_{t+1,n}\in \{S,U{\}}^{2}}{\sum }\alpha_{n}(x_{t,n})\beta_{n}(x_{t+1,n})p(x_{t+1,n}|x_{t,n})p(y_{t+1,n}|x_{t+1,n})}. \end{array}$$

In the maximization step, the new set of parameters can be estimated in a similar way as shown in the previous section. In this case, the expected values are computed using the distributions of all patients. As a result, we obtain the following formulas for the maximization step:

$$\begin{array}{rl} \hat{q} & =\frac{\sum_{n=1}^{N}\sum_{t=0}^{T-1}\xi_{n}(x_{t,n}=S,x_{t+1,n}=U)}{\sum_{n=1}^{N}\sum_{t=0}^{T-1}\gamma_{n}(x_{t,n}=S)},\\ \hat{r} & =\frac{\sum_{n=1}^{N}\sum_{t=0}^{T-1}\xi_{n}(x_{t,n}=U,x_{t+1,n}=U)}{\sum_{n=1}^{N}\sum_{t=0}^{T-1}\gamma_{n}(x_{t,n}=U)},\\ \hat{\;p} & =\frac{\sum_{n=1}^{N}\sum_{t=0}^{T-1}1_{y_{t,n}=1}\gamma_{n}(x_{t,n}=U)}{\sum_{n=1}^{N}\sum_{t=0}^{T-1}\gamma_{n}(x_{t,n}=U)}. \end{array}$$

### 2.4 Patient monitoring policy

The final stage of this method is to devise a policy to decide when the patient is unstable or at a high enough risk of mental health crisis to require close monitoring from the clinical teams. For this, we define a threshold τ above which the patient has a high risk of crisis and needs to be followed closely. To generate our results we used τ=0.35, which maximizes the F1 score in the simulation. This threshold implies an estimated risk of crisis of 35%, but this threshold is adjustable depending on the capacity of the hospital. In order to implement this monitoring policy in clinical practice, we may follow the next steps:

1. Estimate the model parameters for each patient. First, the model parameters for an average patient are estimated using the data from all the patients in the hospital. These model parameters are assigned to all patients that have less than 3 months of data, as they have limited history with the hospital (the minimum number of months is configurable per hospital). The model parameters for the patients with more than 3 months of data are estimated using their individual history of data.
2. Compute the probability of mental health crisis. We can use the estimated parameters of each patient together with the time since their last observed crisis to compute the risk that the patient is going to suffer a mental health crisis during the current week.
3. Decide whether the patient needs close monitoring. If the risk to suffer a mental health crisis is higher than the threshold τ the patient is given close monitoring. Otherwise, the patient is deemed stable and they can be followed through less intensive means.

### 2.5 Data source

The results shown in this paper are based on simulations and an anonymised dataset. This anonymised dataset comprises 4,871 mental health crises from 162 psychiatric patients from the Birmingham and Solihull Mental Health Foundation Trust. The methods described in this manuscript are general and can be applied on similar datasets.

The programming language used to make the simulations, estimate the model parameters and produce the results was Python 3.9.8.

## 3 Results

### 3.1 Policy evaluation with data from a psychiatric hospital

To evaluate the performance of our method with actual data, we applied the steps described in Section 2.4 to a cohort of 162 patients that suffered mental health crises between September of 2012 and August 2016. We divided the dataset into the parameter learning set and the evaluation set. We used the data from 2012 until the end of 2015 to learn the model parameters (parameter learning set) and evaluated the performance of the model using the data from 2016 (evaluation set). The evaluation set corresponds roughly 30% of the data (note that not all patients had their first record during September 2012). The policy threshold was set to 0.35.

We started by estimating the parameters of the average patient following the procedure described at Section 2.3.2. We run the Baum–Welch algorithm with 100 different initial conditions, obtaining $p^{*}=0.95$, $q^{*}=0.037$ and $r^{*}=0.86$ in all of them. This suggests that we reached the global optimum because all the initializations converged to the same model parameters.

**Figure 1:**
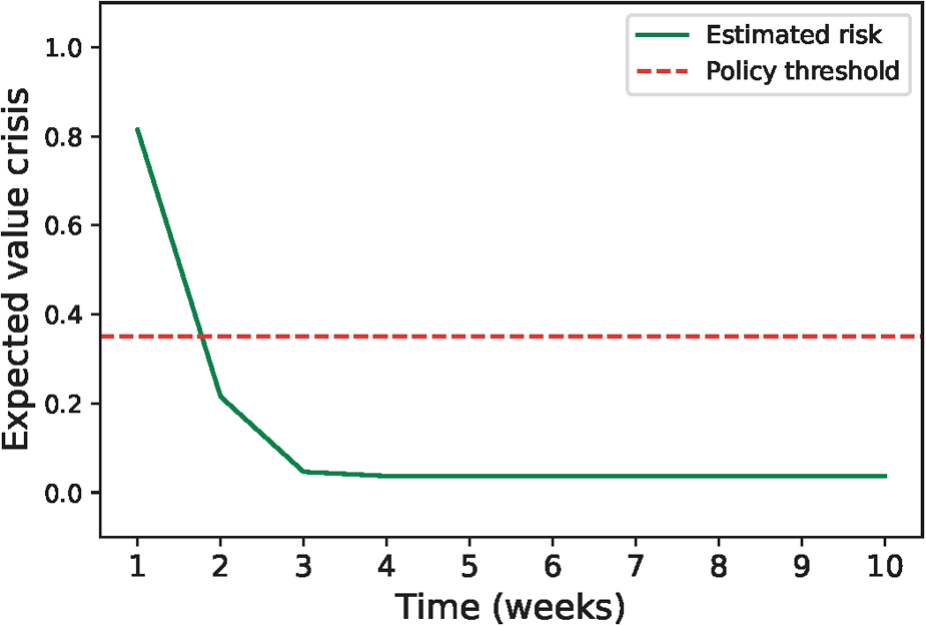
Evolution of the expected value of mental health crisis after the last crisis based on the parameters estimated using all the patients ($q^{*}=0.037$, $r^{*}=0.86$ and $p^{*}=0.95$). The green line shows how the risk of crisis decreases with the number of weeks without crisis and the red line shows the value below which the patient is considered stable.

As shown in Figure 1, with these parameters the risk of mental health crisis starts at 0.81 during the first week after a crisis and decreases each week until the 5th one, when the risk stabilises to 0.037. Under this setting and with the policy threshold at 0.35, the patient should be monitored during the week following a mental health crisis and would be considered to be stable starting the second week after their last crisis.

**Figure 2:**
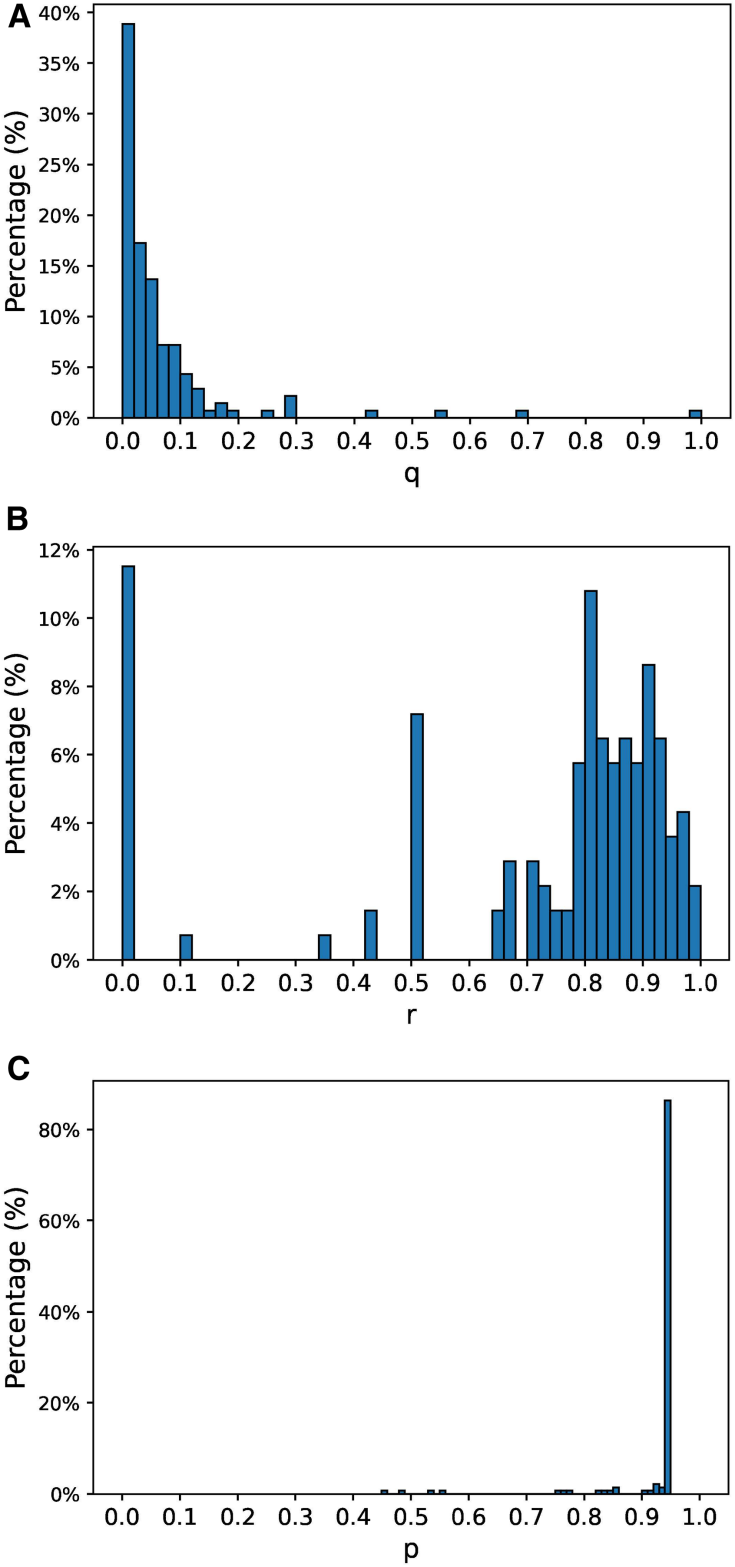
Estimated model parameters’ distributions. (**A**) Distribution of estimated parameter q, (**B**) distribution of estimated parameter r, (**C**) distribution of estimated parameter p.

Then, we estimated the parameters for each patient separately. There were 23 patients (14.1%) that had less than 3 months of data at the end of 2015. The parameters estimated using the complete set of patients were assigned to these patients. For the remaining patients, we individually estimated the model parameters based on their particular observed histories. The distribution of the estimated parameters is shown in Figure 2. The distribution of estimated q is skewed towards 0, denoting that most of the patients have a low probability of relapsing once they are stable. By looking at the distribution of estimated r we see that a large portion of patients (85%) have a probability of staying in an unstable state higher than 0.5. This indicates that most patients tend to stay unstable for more than one week. A significant portion of patients (11.5%) have r=0, which means that these patients usually have isolated crisis and stabilize quickly. Finally, the distribution of estimated p is very skewed towards 1, which means that most patients experience a crisis when they are unstable.

**Figure 3:**
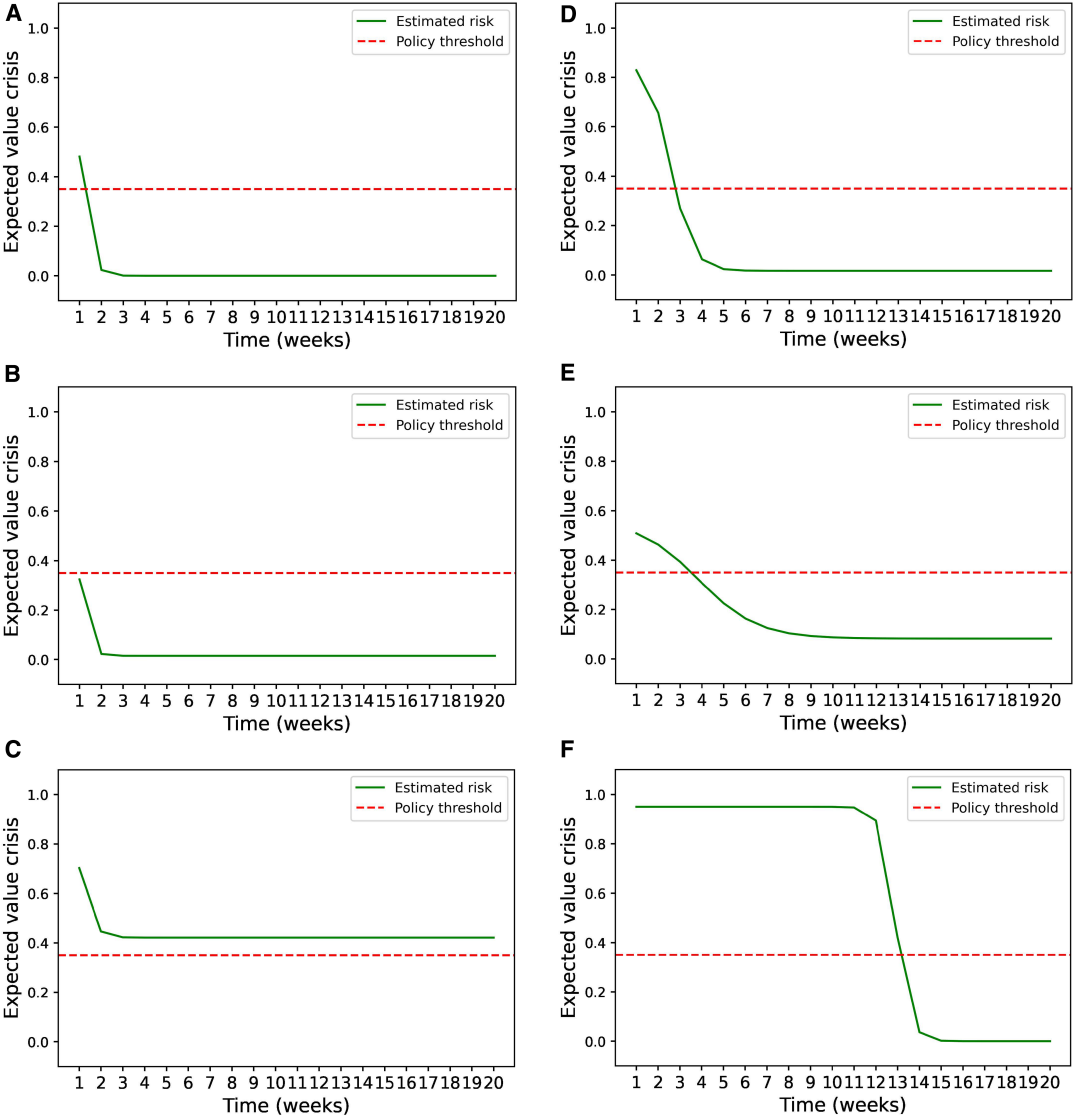
Estimated risk evolution. (**A**–**F**) Examples of how the estimated risk of mental health crisis evolves over the weeks following a mental health crisis for 6 patients that had different model parameters.

The evolution of the estimated risk of mental health crisis over time depends on the patient’s model parameters. Figure 3 shows some examples of how this risk evolves after the patient’s last crisis. Most patients display a pattern similar to A, B and C, having a fast decrease on the estimated risk during the second week after the crisis and reaching convergence to a certain risk level in 3 or 4 weeks. Some other patients, such as examples D, E, had a slower convergence rate that required more than 7 iterations to converge. There were three patients that did not display a significant decrease until 13 weeks after the patient had their last crisis, a representative example is shown in F. These patients exhibit the estimated parameters r and q close to 1 and 0 respectively.

**Figure 4:**
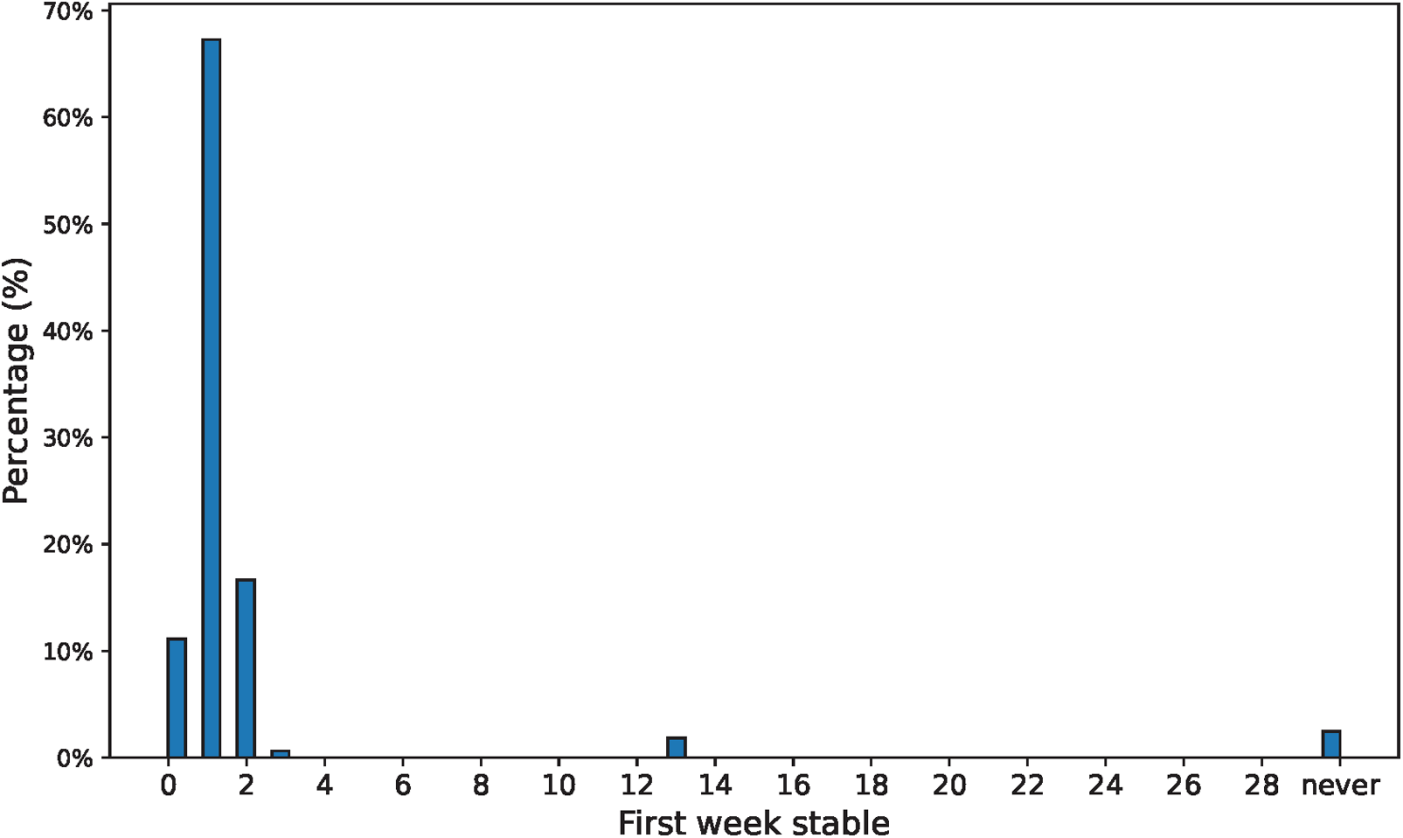
Distribution of the number of weeks that a patient needs to be closely monitored before deemed stable.

In Figure 4, we show the distribution of the number of weeks that the patient needs close monitoring before is considered stable. The proposed policy established that 67.3% of the patients should be closely monitored only one week after their last mental health crisis, 16.7% during the following two weeks and 4.9% for 3 weeks or more (including 2.5% that should be always monitored). The remaining 11.1% were patients whose risk of mental health crisis was lower than the policy threshold at all weeks.

**Figure 5:**
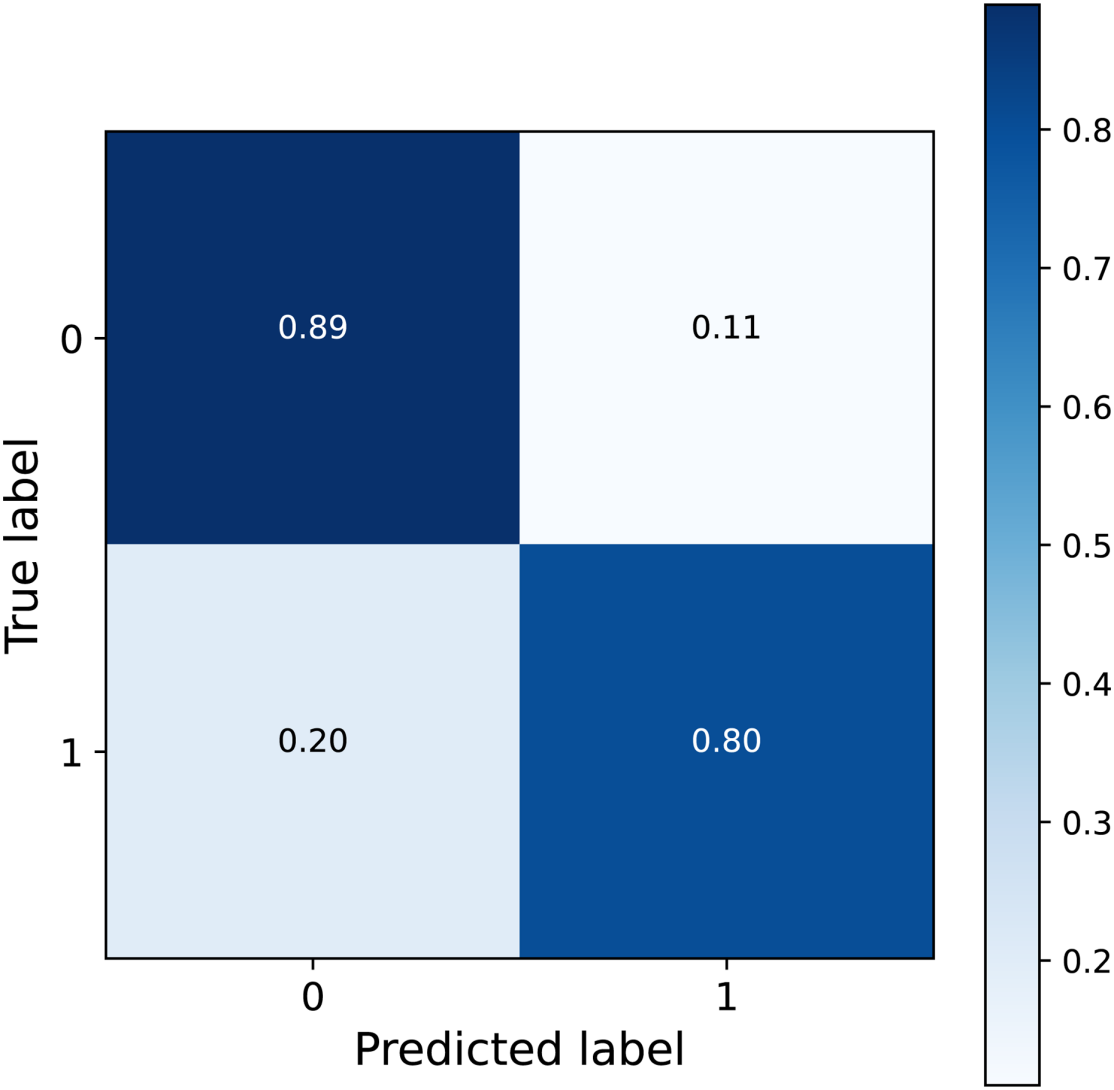
Confusion matrix of the detected crisis by following the policy with a threshold of 0.35.

By following this policy, 56 patients would be closely monitored each week on average (corresponding to 34.3% of the patients) -close to the 55 (33.7%) mental health crisis that occur on average-, among which 78.6% would be patients that suffer a mental health crisis (precision). This policy detects 79.8% of the crises (recall) with a false positive rate of 11.1%, corresponding to a F1-score (22) of 0.79. Figure 5 shows the confusion matrix.

### 3.2 Model learning and policy validation in a hospital simulation

To test how well our method performs given that our assumptions hold, we simulated 5 years of data from a cohort of 3,000 random patients. Each patient has a different set of model parameters generated at random. We chose the distributions to generate the model parameters to resemble the distribution of the estimated model parameters with the data from the psychiatric hospital (see Figure 2). Specifically, we sampled $\tilde{\;p}$, $\tilde{q}$ and $\tilde{r}$ from a lognormal distribution according to

$$\begin{array}{rl} & \tilde{\;p}∼\log N(0,1.2)\\ & \tilde{q}∼\log N(0,1)\\ & \tilde{r}∼\log N(0,0.3). \end{array}$$

**Figure 6:**
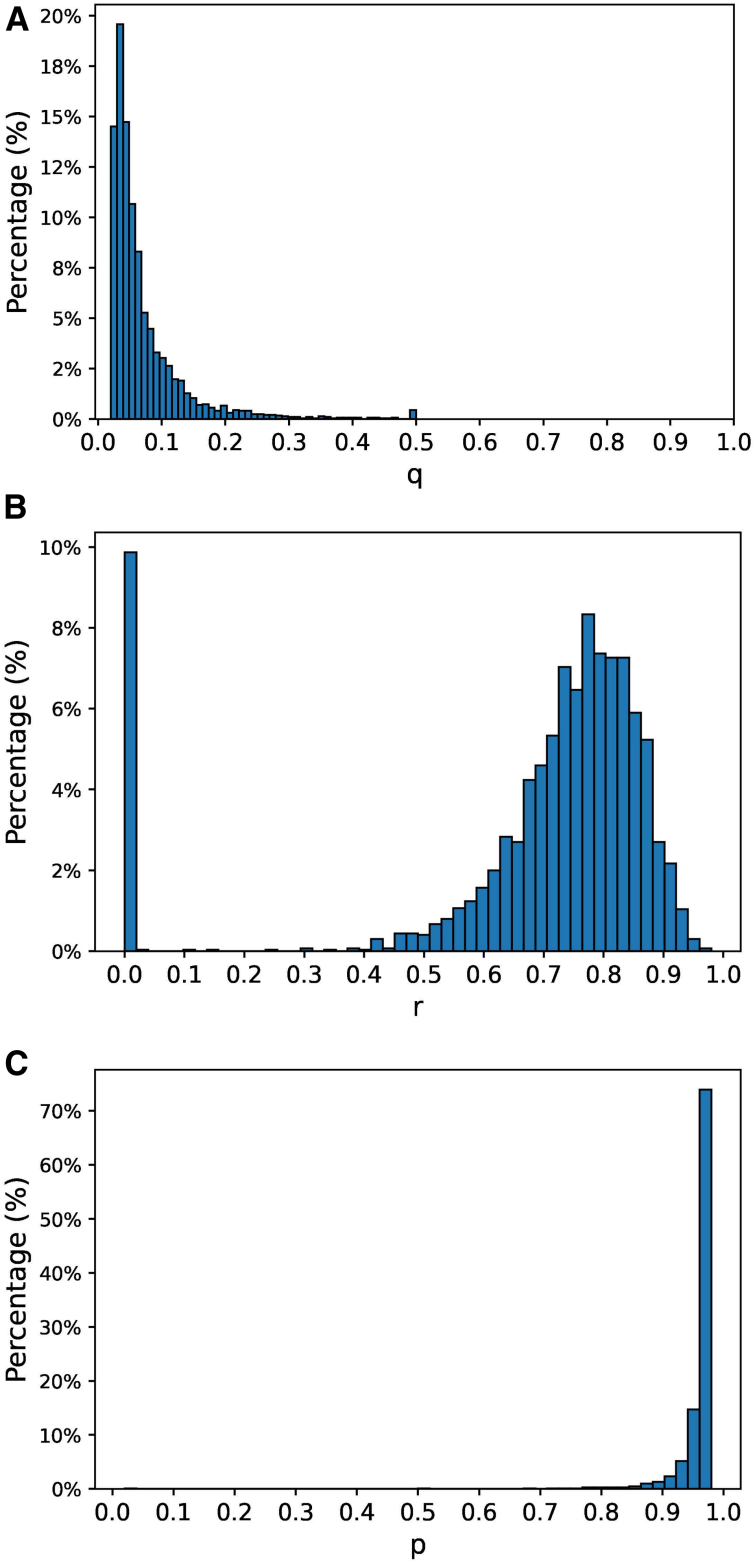
Distribution of the model parameters used in the simulation. (**A**) Distribution of parameter q, (**B**) distribution of parameter r, (**C**) distribution of parameter p.

Then, the values generated by $\tilde{\;p}$, $\tilde{q}$ and $\tilde{r}$ were scaled and transformed to get the p, q and r to lie within the range (0.02,0.98), (0.02,0.5) and (0.02,0.98) respectively. Since we observed that the estimated value of r for around 10% of the patients in the real hospital was 0, we selected 300 patients from the simulation at random and assigned them r=0. The distribution of p, q and r are shown in Figure 6.

**Figure 7:**
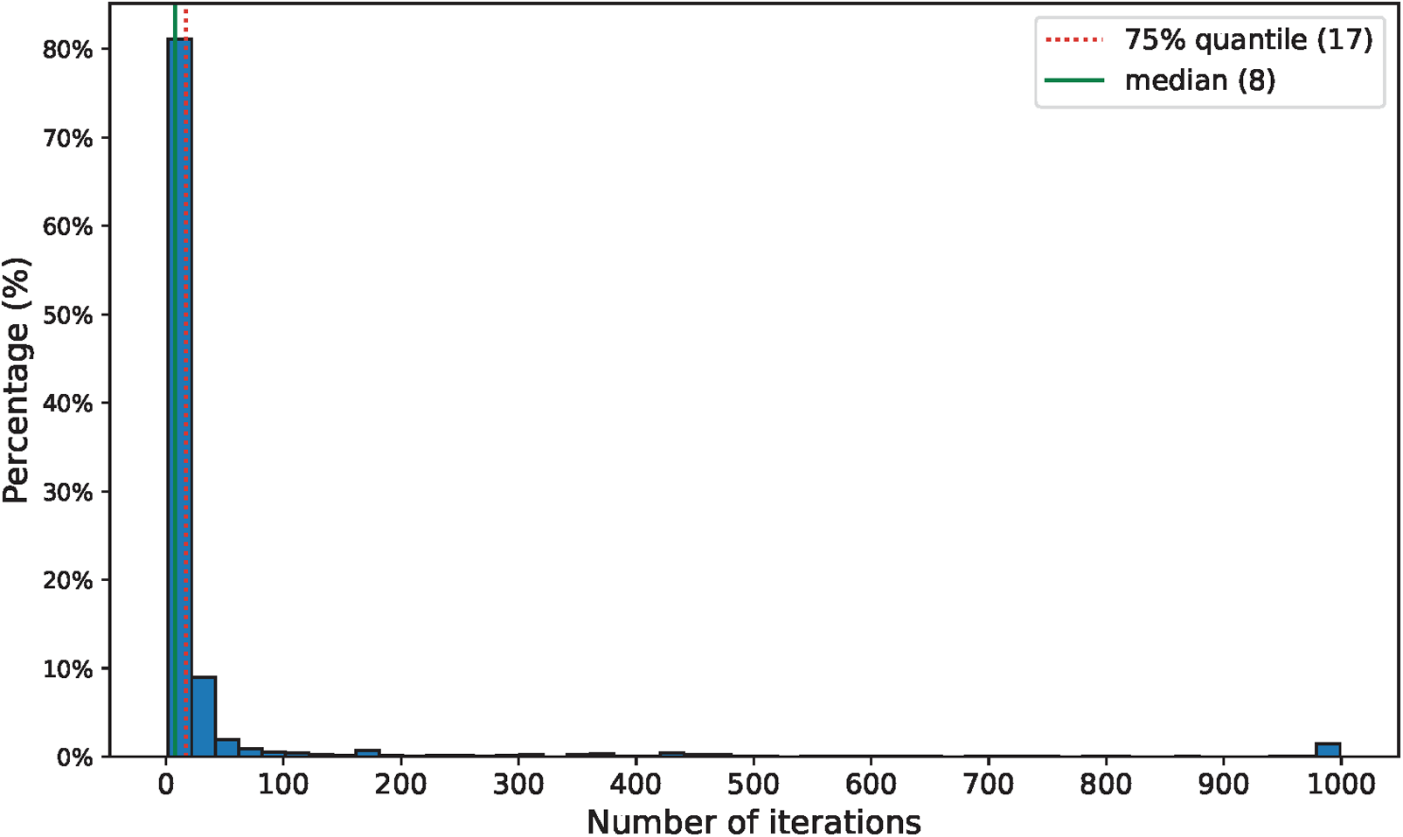
Distribution of the number of iterations required before convergence.

**Figure 8:**
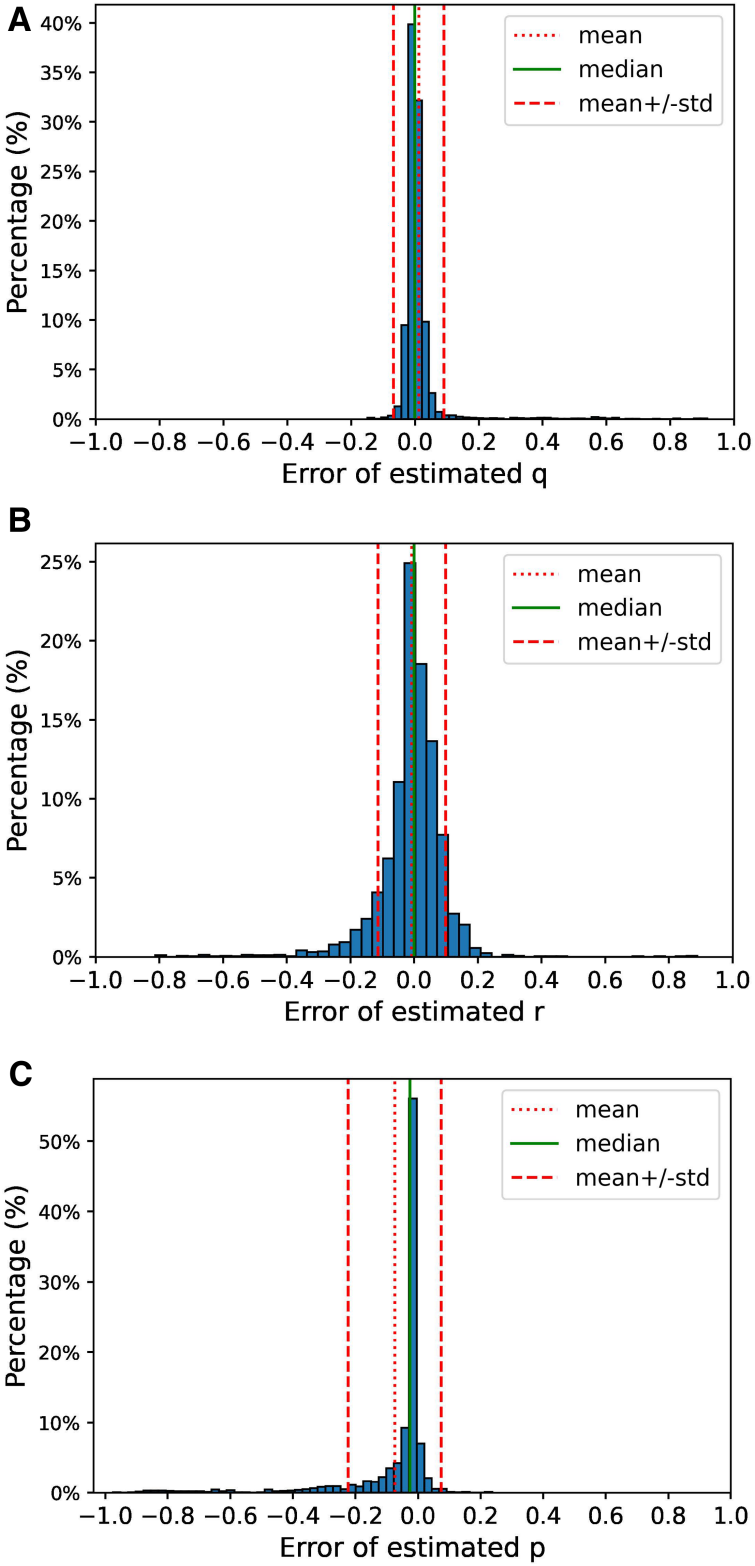
Distribution of the errors during the parameter estimation using the data from the simulation. The error for each of the parameters is computed as estimated parameter minus the actual value of the parameter. (**A**) Distribution of estimation error for parameter q, (**B**) distribution of estimation error for parameter r, (**C**) distribution of estimation error for parameter p.

For each of the patients, we estimated the model parameters by following the steps described in Section 2.3.1 using the data from the first 4 years (parameter learning set). We executed the Expectation and Maximization steps iteratively until convergence or until 1000 iterations were completed. Convergence was defined as the point at which the difference in log-likelihood between two consecutive iterations was less than $10^{-5}$. Remarkably, convergence was achieved in 98.5% of the patients, requiring no more than 50 iterations in 91.4% of the cases. The distribution of iteration counts leading to convergence is shown in Figure 7. The parameter estimation process had a mean absolute error of 0.03 for the parameter q, 0.06 for the parameter r and 0.08 for parameter p. The distribution of the errors is shown in Figure 8.

**Figure 9:**
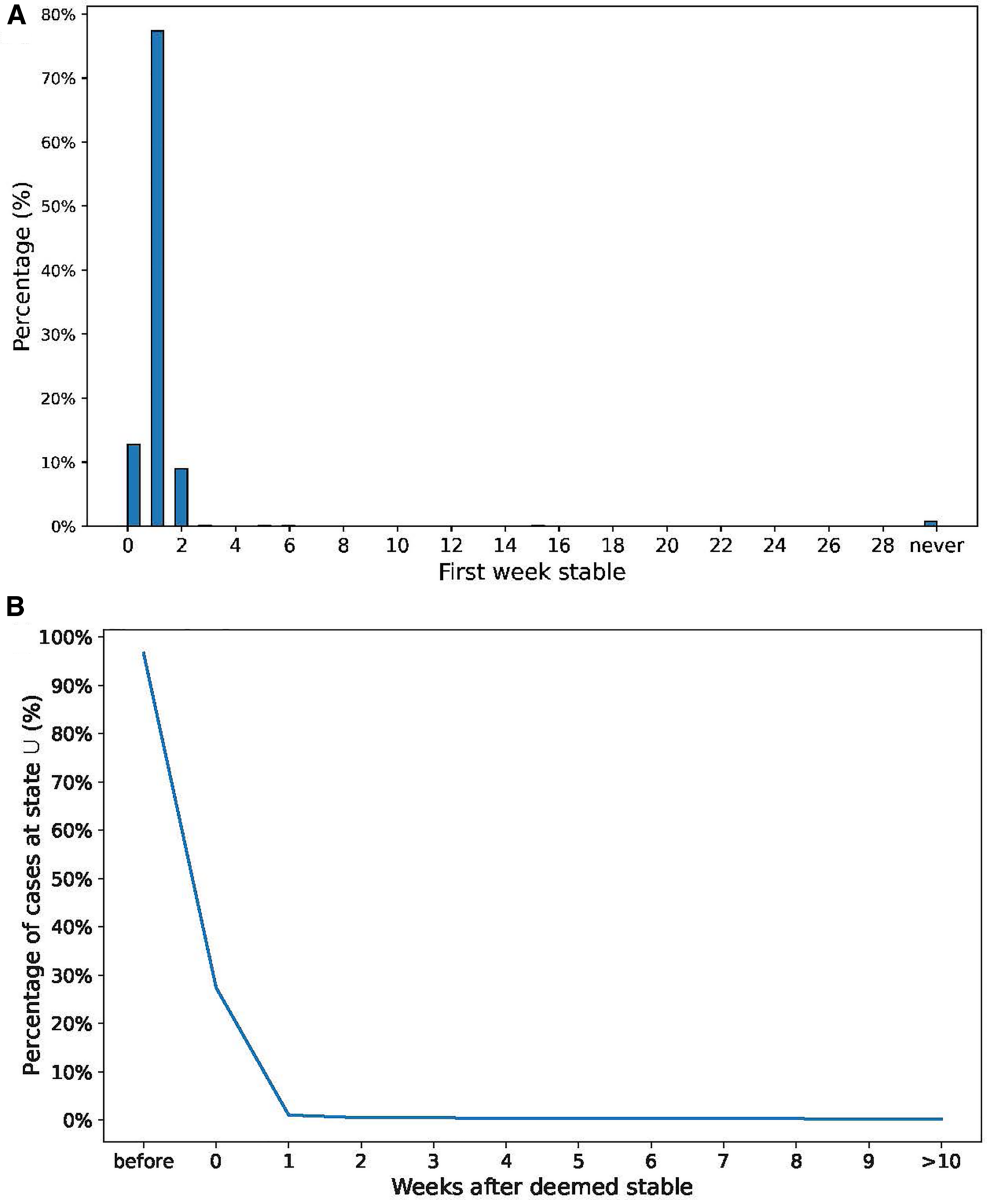
(**A**) Distribution of weeks required to consider the patient “stable”. (**B**) Percentage of U state at each week after the patient is “stable”.

The estimated model parameters were then used to produce the predicted risk of mental health crisis using the Equation 1 from Theorem 1. We computed the predictions for every patient and every week of the last year of the simulation (evaluation set). For the purpose of this simulation, we decided that the policy threshold from which the patients would be considered to be at a high risk of suffering a mental health crisis was 0.35, which corresponded to the maximum F1-score (22) (0.74) in the evaluation set. With this threshold, 77.3% of the patients were considered stable after a week of not having a mental health crisis, 9.0% required 2 weeks to be deemed stable, while 12.7% were estimated to not need close attention even the first week after the mental health crisis occurred. The rest of the patients (1.0%) required 3 or more weeks without a mental health crisis before they are considered to be stable (0.2%) or were considered always unstable (0.8%). Figure 9A shows the distribution of the number of weeks without crisis before a patient is deemed stable. Under this policy there are on average 608 patients at risk of suffering a mental health crisis that should be closely monitored each week, corresponding to 20.3% of the total number of patients. In the same period of time, there were 602 patients on average that suffered a mental health crisis (corresponding to 20.0%), which is close to the number of flagged patients. Among the cases in which a patient was flagged to be monitored closely, 96.2% were patients at state U. In comparison, the patient was at state U only in 25.1% of the cases at the week that the patient was deemed stable and 6.5% of the instances during the following 4 weeks. Figure 9B shows the percentage of patients at state U at each week after the one they were considered to be stable.

## 4 Discussion

In this work, we introduced a novel method to determine the optimal monitoring duration for a psychiatric patient following a mental health crisis before being considered stable. Our method leverages a probabilistic framework utilizing a HMM that solely relies on the historical record of observed crises. To estimate the parameters of the HMM we employed the Baum–Welch algorithm, a well-established technique that remains as the preferred choice to optimize the parameters of a HMM. These parameters can be used to infer the probability that a patient is unstable during the weeks following a mental health crisis and to estimate the risk of a new crisis occurring at each week. Through the resolution of a Ricatti difference equation we demonstrated the existence of a closed-form solution that exhibits exponential convergence and estimated the probability of a mental health crisis at each week following the last occurrence. These results enable the development of a policy for determining the point at which a patient can be deemed stable, with a minimal risk of experiencing a new mental health crisis.

When defining the probabilistic model for mental health states, we established four assumptions. First, we assumed that patients can be in one of two possible mental states during any given week: stable (S) or unstable (U). These states evolve following a Markov Chain, providing a simplified framework that reduces the complexity of the model and makes it more tractable for analysis. Another key assumption is that the patient’s mental state remains hidden and is only partially observable through the crisis variable. This assumption reflects the practical constraints associated with directly measuring a patient’s mental state. It aligns with real-world scenarios, where a patient’s state is indirectly inferred through interactions with the hospital. Furthermore, we assumed that patients in state S cannot experience a crisis, while those at state U have a non-zero probability of suffering a crisis. This assumption aligns with reality and simplifies the modeling of crisis events by directly linking them to the patient’s current mental state. Finally, we assumed that p<1 and q≠r as it enables the solution of the Riccati equation presented in Section 2.2. This is a reasonable assumption because patients in an unstable state do not experience crises continuously until they stabilize, and patients are often more likely to remain in their current state than to switch (typically, r>q).

The assumptions we made serve the purpose of simplifying a complex problem, making it tractable for analysis, all while maintaining consistency with real-world scenario. However, it is essential to acknowledge potential limitations. Firstly, the evolution of a patient’s mental state is a complex process and our model may not fully capture the spectrum of mental states a patient can experience or the intricacy of their transitions. To address this, the model could be extended by introducing a broader range of possible states and considering a higher order Markov Chain (23), which accounts for the influence of past mental states. Although this would yield different analytical results in Section 2.2, similar steps could be taken, and an adapted version of the Baum–Welch algorithm could be applied to estimate the model parameters in higher order HMM (24). However, the introduction of additional parameters to the model would make the parameter estimation harder. Secondly, we assumed that the probability that a patient in state U suffers a mental health crisis remains constant, yet this probability might increase or decrease over time in state U. Introducing a time dependence to the variable p would not alter the solution from Theorem 1, but the parameter estimation would change based on the chosen family of functions used to define this time dependence. Finally, while mental health crises are the sole observed signal in our model, clinicians may directly or indirectly observe the mental state of a patient during regular visits. Theorem 1 provides the solution when an S state is observed, and the inclusion of these observations would enhance the estimation of the model parameters. However, the incorporation of additional relevant information, such as data from routine visits between crises, diagnosed disorders, or prescribed medications, would require further research.

We presented two sets of results. The first set, based on actual data collected at a psychiatric hospital, aimed to assess the performance of our method in a real-world scenario. When we estimated the model parameters assuming uniform model parameters for all patients, the predicted risk of mental health crisis dropped substantially between the first and the second week after the last mental health crisis, from 0.81 to 0.21. This suggests that by employing a one-size-fits-all approach, patients can generally be considered stable after just one week, aligning with previous literature assumptions (15, 16). However, when we estimated the parameters individually for each patient, significant variations emerged. In most cases, $p^{*}$ exceeded 0.9, but $r^{*}$ ranged from 0 in 11.5% of the cases to skewing towards 1 in the remainder, while $q^{*}$ predominantly skewed towards 0. Each patient’s risk of crisis exhibited distinct patterns based on their estimated parameters. Under our policy, 67.3% of the patients required close monitoring only during the first week after their last mental health crisis, 16.7% for two weeks, and 4.9% for three weeks or more. A small percentage of patients (2.5%) maintained a risk above 0.35 even after convergence, requiring continuous close monitoring. In contrast, 11.1% of patients never exceeded and did not necessitate monitoring according to our policy. The application of this policy yielded an F1 score of 0.79 in the evaluation set, detecting 79.8% of the crises with a false positive rate of 11.1%. While these outcomes underscore the method’s strong performance with real-world data and its potential to determine the optimal monitoring duration for each patient before deeming them stable, it is essential to note that these conclusions are drawn from a sample size of 162. This limitation suggests the importance of further validation with larger patient cohorts.

The second set of results, based on a simulation designed to validate the performance of our method when our underlying assumptions are met. In this analysis, we observed rapid convergence in the parameter estimation step across nearly all the cases, with a mean absolute error below 0.1 for all three estimated parameters. However, it is important to note that in a small number of instances, substantial differences emerged between our estimated parameters and their actual values. Leveraging these estimated model parameters, we predicted the risk of mental health crisis as outlined in Theorem 1 and created a policy to identify those patients with a risk exceeding a defined threshold (0.35 in this particular case). By applying this policy, we obtained an F1 score of 0.74, with 96.2% of the flagged patients in an unstable state. These results provide compelling evidence that our method performs as intended when our model assumptions hold.

Recurring mental health crises pose a profound threat to both the individual patient’s mental and social well-being, and by extension, that of their family. With each hospital admission, a substantial allocation of resources becomes imperative, imposing a considerable financial burden on mental healthcare facilities. While hospitals typically implement one-size-fits-all policies shaped by the needs of the majority of patients, these policies often overlook the nuanced variations in mental health disorders and among individual patients. Our innovative data-driven approach offers a bespoke assessment that meticulously considers these variations, paving the way for more personalized and effective mental healthcare interventions.

Recurring mental health crises pose a profound threat to both the individual patient’s mental and social well-being, and by extension, that of their family. With each hospital admission, a large quantity of resources needs to be allocated to treat the patient, imposing a considerable financial burden on mental healthcare facilities. While hospitals typically implement one-size-fits-all policies driven by the needs of the majority of patients, these policies often overlook the nuanced variations in mental health disorders and among individual patients. Our data-driven approach provides an individualized assessment that considers these variations, paving the way for more personalized and effective mental healthcare interventions.

## Data Availability

The datasets presented in this article are not readily available because hospital data cannot be shared publicly due to the risk of violating privacy. All the other sources including the code used to generate the simulated data, estimate the model parameters, evaluate the models and generate the results presented in this study can be found at the open source repository https://github.com/Icedgarr/post_crisis_monitoring. Requests to access the datasets should be directed to Roger Garriga, roger.garrigacalleja@koahealth.com.
