# Supplementary material for: Individualized post-crisis monitoring of psychiatric patients via Hidden Markov models
Source: Front Digit Health. 2024 Feb 2;6:1322555. doi: 10.3389/fdgth.2024.1322555 (PMC10869627; doi:10.3389/fdgth.2024.1322555)
Supplement: Supplementary file 1 [file Presentation1.pdf]

APPENDIX A: PROOF OF THE LEMMAS

In this Appendix we provide the proofs of the Lemmas needed to prove Theorem 1 and the Corollary that follows the theorem.

THEOREM 1. *Let $\mathcal{O}_{t-1} = \{X_0 = x_0, Y_0 = y_0, Y_1 = 0, \ldots, Y_{t-1} = 0\}$ be the observed history of a patient n up to the week t and p, q, r the model parameters associated with the patient. Then, the probability that patient n suffers a mental health crisis at week t is given by*

$$P(Y_t = 1|\mathcal{O}_{t-1}) = 1 - (1 - pr + r - q)\frac{(y_0 - y_-)y_+^{t+1} - (y_+ - y_0)y_-^{t+1}}{(y_0 - y_-)y_+^{t} - (y_+ - y_0)y_-^{t}}, \tag{1}$$

with

$$\begin{aligned} y_+ &= \frac{1 + \sqrt{1 - 4\frac{(r-q)(1-p)}{(1-pr+r-q)^2}}}{2}, \\ y_- &= \frac{1 - \sqrt{1 - 4\frac{(r-q)(1-p)}{(1-pr+r-q)^2}}}{2}, \\ y_0 &= \frac{2Rw_{x_0} - R}{2R + w_{x_0} - 1}, \end{aligned}$$

where $R = \frac{(r-q)(1-p)}{(1-pr+r-q)^2}$, $w_U = \frac{1-pr}{1-pr+r-q}$ (when $x_0 = U$) and $w_S = \frac{1-pq}{1-pr+r-q}$ (when $x_0 = S$).

To prove Theorem 1 we need the following lemmas:

LEMMA 1. *Let $\mathcal{O}_{t-1} = \{X_0 = x_0, Y_0 = y_0, Y_1 = 0, \ldots, Y_{t-1} = 0\}$ be the observed history of a patient n up to the week t and p, q, r the model parameters associated with the patient. Then, the probability that the patient is at state U at week t is given by a first order rational difference equation, also called Riccati difference equation (1), of the form $w_{t+1} = \frac{aw_t+b}{cw_t+d}$ where $a = r - q - pr$, $b = q$, $c = -p$ and $d = 1$. Hence,*

$$P(X_t = U|\mathcal{O}_{t-1}) = \frac{q + (r - q - pr)P(X_{t-1} = U|\mathcal{O}_{t-2})}{1 - pP(X_{t-1} = U|\mathcal{O}_{t-2})}. \tag{2}$$

PROOF. We start by writing $P(X_t = U|\mathcal{O}_{t-1})$ as a function of $P(X_{t-1} = U|\mathcal{O}_{t-1})$:

$$\begin{aligned} P(X_t = U|\mathcal{O}_{t-1}) &= P(X_t = U, X_{t-1} = S|\mathcal{O}_{t-1}) + P(X_t = U, X_{t-1} = U|\mathcal{O}_{t-1}) \\ &= P(X_t = U|X_{t-1} = S, \mathcal{O}_{t-1})P(X_{t-1} = S|\mathcal{O}_{t-1}) + \\ &\quad + P(X_t = U|X_{t-1} = U, \mathcal{O}_{t-1})P(X_{t-1} = U|\mathcal{O}_{t-1}) \text{ (by the Markov property)} \\ &= qP(X_{t-1} = S|\mathcal{O}_{t-1}) + rP(X_{t-1} = U|\mathcal{O}_{t-1}) \\ &= q(1 - P(X_{t-1} = U|\mathcal{O}_{t-1})) + rP(X_{t-1} = U|\mathcal{O}_{t-1}) \\ &= q + (r - q)P(X_{t-1} = U|\mathcal{O}_{t-1}). \end{aligned}$$

Then, we express $P(Y_{t-1} = 0|Y_1^{t-2} = 0, X_0 = U)$ as a function of $P(X_{t-1} = U|Y_1^{t-2} = 0, X_0 = U)$ as we will need the result later:

$$
\begin{aligned}
&P(Y_{t-1} = 0|Y_1^{t-2} = 0, X_0 = U) = \\
&= P(Y_{t-1} = 0, X_{t-1} = S|Y_1^{t-2} = 0, X_0 = U) + P(Y_{t-1} = 0, X_{t-1} = U|Y_1^{t-2} = 0, X_0 = U) \\
&= P(Y_{t-1} = 0|X_{t-1} = S)P(X_{t-1} = S|Y_1^{t-2}, X_0 = U)+ \\
&\quad + P(Y_{t-1} = 0|X_{t-1} = U)P(X_{t-1} = U|Y_1^{t-2}, X_0 = U) \qquad (3) \\
&= 1 \cdot (1 - P(X_{t-1} = U|Y_1^{t-2}, X_1 = U)) + (1-p)P(X_{t-1} = U|Y_1^{t-2}, X_0 = U) \\
&= 1 - pP(X_{t-1} = U|Y_1^{t-2} = 0, X_0 = U).
\end{aligned}
$$

Now, we can use the Bayes Theorem and the previous result to get the recursion

$$
\begin{aligned}
&P(X_t = U|\mathcal{O}_{t-1}) = q + (r-q)P(X_{t-1} = U|Y_1^{t-1} = 0, X_0 = U) \\
&= q + (r-q)P(X_{t-1} = U|Y_{t-1} = 0, Y_1^{t-2} = 0, X_0 = U) \\
&= q + (r-q)\frac{P(Y_{t-1} = 0|X_{t-1} = U, Y_1^{t-2} = 0, X_0 = U)P(X_{t-1} = U|Y_1^{t-2} = 0, X_0 = U)}{P(Y_{t-1} = 0|Y_1^{t-2} = 0, X_0 = U)} \\
&= q + (r-q)\frac{(1-p)P(X_{t-1} = U|Y_1^{t-2} = 0, X_0 = U)}{1 - pP(X_{t-1} = U|Y_1^{t-2} = 0, X_0 = U)} \text{ (by 3)} \\
&= \frac{q - pqP(X_{t-1} = U|Y_1^{t-2} = 0, X_0 = U) + (r-q)(1-p)P(X_{t-1} = U|Y_1^{t-2} = 0, X_0 = U)}{1 - pP(X_{t-1} = U|Y_1^{t-2} = 0, X_0 = U)} \\
&= \frac{q + ((r-q)(1-p) - pq)P(X_{t-1} = U|Y_1^{t-2} = 0, X_0 = U)}{1 - pP(X_{t-1} = U|Y_1^{t-2} = 0, X_0 = U)} \\
&= \frac{q + (r - q - pr)P(X_{t-1} = U|Y_1^{t-2} = 0, X_0 = U)}{1 - pP(X_{t-1} = U|Y_1^{t-2} = 0, X_0 = U)} \\
&= \frac{q + (r - q - pr)P(X_{t-1} = U|\mathcal{O}_{t-2})}{1 - pP(X_{t-1} = U|\mathcal{O}_{t-2})}.
\end{aligned}
$$

□

LEMMA 2. *For all q, r, p such that $0 \leq q \leq 1$, $0 \leq r \leq 1$ and $0 \leq p \leq 1$, we have*

$$\frac{(r-q)(1-p)}{(1 - pr + r - q)^2} \leq \frac{1}{4}.$$

PROOF. If $q > r$, the left-hand side is negative, hence less than $\frac{1}{4}$. To show that the inequality holds if $r > q$ we define $x = r - q$, $0 < x < 1$ and develop the inequality:

$$\begin{aligned}
&\frac{x(1-p)}{(1-pr+x)^2} \le \frac{1}{4} && \iff \\
&4x - 4px \le p^2r^2 - 2prx - 2pr + x^2 + 2x + 1 && \iff \\
&0 \le r^2p^2 + (4x - 2rx - 2r)p + x^2 - 2x + 1 && \iff \\
&r^2p^2 + (4x - 2rx - 2r)p + (x-1)^2 \ge 0. &&
\end{aligned}$$

The left-hand side of the inequality is quadratic in p with a positive coefficient in the second degree term which implies that the function is convex in p and has a minimum as long as $r^2 \neq 0$. To find the minimum, we find the value where its derivative is 0.

$$2pr^2 + 2(2x - r - rx) = 0 \iff p = \frac{rx + r - 2x}{r^2}.$$

By substituting in the inequality we obtain

$$\begin{aligned}
&\frac{(rx+r-2x)^2}{r^2} + \frac{2(2x - rx - r)(rx + r - 2x)}{r^2} + (x-1)^2 \ge 0 && \iff \\
&\frac{(rx+r-2x)^2}{r^2} - 2\frac{(rx+r-2x)^2}{r^2} + (x-1)^2 \ge 0 && \iff \\
&(x-1)^2 - \frac{(rx+r-2x)^2}{r^2} \ge 0 && \iff \\
&r^2(x^2 - 2x + 1) - (r^2x^2 + 2r^2x - 4rx^2 + r^2 - 4rx + 4x^2) \ge 0 && \iff \\
&r^2x^2 - 2r^2x + r^2 - r^2x^2 - 2r^2x + 4rx^2 - r^2 + 4rx - 4x^2 \ge 0 && \iff \\
&4rx^2 + 4rx - 4r^2x - 4x^2 \ge 0 && \iff \\
&4x(rx + r - r^2 - x) \ge 0 && \iff \\
&x(r-1)(x-r) \ge 0 && \iff \\
&x(1-r)(r - (r-q)) \ge 0 && \iff \\
&x(1-r)q \ge 0
\end{aligned}$$

Since $x \ge 0$, $1 - r \ge 0$ and $q \ge 0$, the inequality holds, so the roots of the characteristic equation are real for all $0 \le p < 1$, $0 \le q \le 1$ and $0 \le r \le 1$ given that $q \neq r$.

□

Using the results from Lemmas 1 and 2, we can prove Theorem 1:

PROOF. The optimal solution to estimate the risk that patient n suffers a mental health crisis at week t given the observed history $\mathcal{O}_{t-1} = \{X_0 = x_0, Y_0 = y_0, Y_1 = 0, \ldots, Y_{t-1} = 0\}$ is computed as

$$P(Y_t = 1|\mathcal{O}_{t-1}) = pP(X_t = U|\mathcal{O}_{t-1}).$$

We can compute the result by recursion, expressing $P(X_t = U|\mathcal{O}_{t-1}) = P(X_t = U|Y_1^{t-1} = 0, X_0 = U)$ as a function of $P(X_t = U|\mathcal{O}_{t-2}) = P(X_{t-1} = U|Y_1^{t-2} = 0, X_0 = U)$ and solving the recurrence function. By Lemma 1 we obtain the Riccati difference equation in Equation 2 to compute $P(X_t = U|\mathcal{O}_{t-1})$. A Riccati difference equation when $ad - bc \neq 0$ can be solved by transforming the sequence w_t into a linear recurrence relation through the transformed sequences $y_{t+1} = 1 - \frac{R}{y_t}$ and $y_t = \frac{x_{t+1}}{x_t}$, where $R = \frac{ad-bc}{(a+d)^2}$ and $w_t = \frac{a+d}{c} y_t - \frac{d}{c}$ (1). This transformation yields the linear recurrence $x_{t+2} - x_{t+1} + R x_t = 0$ for $y_t = \frac{x_{t+1}}{x_t}$(1).

First we show which conditions need to be met for $ad - bc \neq 0$:

$$ad - cb = r - q - pr + pq = r - q - p(r - q) = (r - q)(1 - p).$$

This implies that $ad - bc \neq 0 \iff q \neq r$ and $p \neq 1$, which holds as part of the assumptions. Now, we may solve the linear recurrence relation:

$$R = \frac{ad - bc}{(a + d)^2} = \frac{(r - q)(1 - p)}{(1 - pr + r - q)^2},$$

$$x^2 - x + \frac{(r - q)(1 - p)}{(1 - pr + r - q)^2} = 0.$$

Using the formula to solve the second-order equation, we obtain the roots

$$\frac{1 \pm \sqrt{1 - 4\frac{(r-q)(1-p)}{(1-pr+r-q)^2}}}{2}.$$

The roots are real if and only if $\frac{(r-q)(1-p)}{(1-pr+r-q)^2} \leq \frac{1}{4}$. By Lemma 2 this inequality holds for all q, r, p such that $0 \leq q \leq 1$, $0 \leq r \leq 1$ and $0 \leq p \leq 1$. As shown in (1), the solution of the transformed sequence y_t is :

$$y_t = \frac{(y_0 - y_-)y_+^{t+1} - (y_+ - y_0)y_-^{t+1}}{(y_0 - y_-)y_+^t - (y_+ - y_0)y_-^t},$$

with $y_- = \frac{1 - \sqrt{1 - 4\frac{(r-q)(1-p)}{(1-pr+r-q)^2}}}{2}$, $y_+ = \frac{1 + \sqrt{1 - 4\frac{(r-q)(1-p)}{(1-pr+r-q)^2}}}{2}$. From the definition of y_t described above, we have

$$P(X_t = U|\mathcal{O}_{t-1}) = \frac{a + d}{c} y_t - \frac{d}{c} = -\frac{1 - pr + r - q}{p} \frac{(y_0 - y_-)y_+^{t+1} - (y_+ - y_0)y_-^{t+1}}{(y_0 - y_-)y_+^t - (y_+ - y_0)y_-^t} + \frac{1}{p},$$

which implies that the probability of crisis is

$$P(Y_t = 1|\mathcal{O}_{t-1}) = p\left(\frac{1}{p} - \frac{1-pr+r-q}{p}\frac{(y_0-y_-)y_+^{t+1}-(y_+-y_0)y_-^{t+1}}{(y_0-y_-)y_+^{t}-(y_+-y_0)y_-^{t}}\right) \tag{4}$$
$$= 1-(1-pr+r-q)\frac{(y_0-y_-)y_+^{t+1}-(y_+-y_0)y_-^{t+1}}{(y_0-y_-)y_+^{t}-(y_+-y_0)y_-^{t}},$$

The last step to prove Theorem 1 is to find y_0. First, we start by computing y_0 when $x_0 = U$. Since $P(X_1 = U|Y_0 = 0, X_0 = U) = P(X_1 = U|X_0 = U) = r$, we can use the equivalence in Equation 4 with $t = 1$:

$$r = \frac{1}{p} - \frac{1-pr+r-q}{p}\frac{(y_0-y_-)y_+^{2}-(y_+-y_0)y_-^{2}}{(y_0-y_-)y_+-(y_+-y_0)y_-} \iff$$
$$\frac{y_0y_+^2 - y_-y_+^2 - y_+y_-^2 + y_0y_-^2}{y_0y_+ - y_-y_+ - y_+y_- + y_0y_-} = \frac{1-pr}{1-pr+r-q}$$

To simplify, we denote the right-hand of the equation by w_U, i.e., $w_U = \frac{1-pr}{1-pr+r-q}$. Notice that when $x_0 = S$ $P(X_1 = U|Y_0 = 0, X_0 = S) = P(X_1 = U|X_0 = S) = q$, thus we obtain $w_S = \frac{1-pq}{1-pr+r-q}$. Given that the only factor that depends on x_0 is w_{x_0} we keep it general in the following steps of the proof. Now, we isolate the y_0

$$\frac{y_0y_+^2 - y_-y_+^2 - y_+y_-^2 + y_0y_-^2}{y_0y_+ - y_-y_+ - y_+y_- + y_0y_-} = w_{x_0} \iff$$
$$y_0y_+w_{x_0} - 2y_-y_+w_{x_0} + y_0y_-w_{x_0} = y_0y_+^2 - y_-y_+^2 - y_+y_-^2 + y_0y_-^2 \iff$$
$$y_0(y_+w_{x_0} + y_-w_{x_0} - y_+^2 - y_-^2) = 2y_-y_+w_{x_0} - y_-y_+^2 - y_+y_-^2 \iff$$
$$y_0 = \frac{2y_-y_+w_{x_0} - y_-y_+^2 - y_+y_-^2}{y_+w_{x_0} + y_-w_{x_0} - y_+^2 - y_-^2} \iff$$
$$y_0 = \frac{2Rw_{x_0} - \frac{1}{2}R(1+\sqrt{1-4R}) - \frac{1}{2}R(1-\sqrt{1-4R})}{w_{x_0} - \frac{1}{2}(1-2R+\sqrt{1-4R}) - \frac{1}{2}(1-2R-\sqrt{1-4R})} \iff$$
$$y_0 = \frac{2Rw_{x_0} - R}{2R + w_{x_0} - 1}.$$

To get from the 4th to the 5th step we used the following expressions:

$$
\begin{aligned}
y_+^2 &= \frac{1}{4}(1+1-4R+2\sqrt{1-4R}) = \frac{1}{2}(1-R+\sqrt{1-4R}) \\
y_-^2 &= \frac{1}{4}(1+1-4R-2\sqrt{1-4R}) = \frac{1}{2}(1-R-\sqrt{1-4R}) \\
y_+y_- &= y_-y+ = \frac{1}{4}(1-(1-4R)) = R \\
y_+y_-^2 &= \frac{1}{8}(4R)(1-\sqrt{1-4R}) = \frac{1}{2}R(1-\sqrt{1-4R}) \\
y_-y_+^2 &= \frac{1}{8}(4R)(1+\sqrt{1-4R}) = \frac{1}{2}R(1+\sqrt{1-4R}) \\
y_+ + y_- &= \frac{1}{2}(1+\sqrt{1-4R})\frac{1}{2}(1-\sqrt{1-4R}) = 1.
\end{aligned}
$$

□

Corollary 1.1 directly follows from Theorem 1.

COROLLARY 1.1. *The optimal solution to estimate the risk that a patient with model parameters* q, r, p *converges to* $1-(1-pr+r-q)y_+$ *when* t *grows.*

PROOF. Since the optimal solution is given by Equation 1 and $y_+ > y_-$, we have

$$
\begin{aligned}
\lim_{t\to\infty} P(Y_t = 1|\mathcal{O}_{t-1}) &= \lim_{t\to\infty}\left(1-(1-pr+r-q)\frac{(y_0-y_-)y_+^{t+1}-(y_+-y_0)y_-^{t+1}}{(y_0-y_-)y_+^t-(y_+-y_0)y_-^t}\right) \\
&= \lim_{t\to\infty}\left(1-(1-pr+r-q)\frac{y_+^t(y_0-y_-)y_+ - y_+^t(y_+-y_0)\frac{y_-^{t+1}}{y_+^t}}{y_+^t(y_0-y_-)-y_+^t(y_+-y_0)\frac{y_-^t}{y_+^t}}\right) \\
&= \lim_{t\to\infty}\left(1-(1-pr+r-q)\frac{(y_0-y_-)y_+ - (y_+-y_0)y_-(\frac{y_-}{y_+})^t}{(y_0-y_-)-(y_+-y_0)(\frac{y_-}{y_+})^t}\right) \\
&= \lim_{t\to\infty}\left(1-(1-pr+r-q)\frac{(y_0-y_-)y_+}{(y_0-y_-)}\right) \\
&= 1-(1-pr+r-q)y_+.
\end{aligned}
$$

□

REFERENCES

1 .Kulenovic MR, Ladas G. *Dynamics of second order rational difference equations: with open problems and conjectures* (New York, CRC Press) (2001).
